# Draft Genome Sequence of *Escherichia coli* Strain LCT-EC59

**DOI:** 10.1128/genomeA.00242-12

**Published:** 2013-02-21

**Authors:** Tianzhi Li, Jiapeng Chen, De Chang, Xiangqun Fang, Junfeng Wang, Yinghua Guo, Longxiang Su, Guogang Xu, Yajuan Wang, Zhenhong Chen, Changting Liu

**Affiliations:** Nanlou Respiratory Diseases Department, Chinese PLA General Hospital, Beijing, China

## Abstract

The space environment is a very special condition under which many organisms change many features. *Escherichia coli* is employed widely as a prokaryotic model organism in the fields of biotechnology and microbiology. Here, we present the draft genome sequence of *E. coli* strain LCT-EC59 exposed to space conditions.

## GENOME ANNOUNCEMENT

The space environment, with its microgravity, radiation, and magnetism, might affect microorganisms by changing a variety of characteristics, such as morphology, growth rate, metabolism, virulence, and antibiotic resistance (1–5). Wilson et al., using a murine infection model, reported that spaceflight-grown *Salmonella enterica* presented enhanced virulence and extracellular matrix accumulation associated with a biofilm (6). Moreover, it is reported that during spaceflight missions, *Pseudomonas aeruginosa* presumably adopted an anaerobic mode of growth, in which denitrification was obvious (7). However, the effects of the space environment on *Escherichia coli* remain to be investigated. Here, we describe the draft genome sequence of *E. coli* strain LCT-BC59, derived from an *E. coli* strain (CGMCC 1.2385) that was sent into space for 398 h by the ShenZhou-8 spacecraft from 1 November 2011 to 17 November 2011.

After extracting whole-genomic DNA from the sample, we fragmented DNA with a Covaris E210 ultrasonicator to build 500-bp and 6-kbp libraries. The DNA fragments were purified and then connected with poly(A) tails and adaptors for hybridization with sequencing primers. We performed amplification for the 500-bp and 6-kbp libraries with genome coverage of ∼100× and ∼50×, respectively. We then sequenced 90 bp of both ends of those fragments on Illumina HiSeq 2000, according to the manufacturer’s instructions (8).

After base calling, sequenced reads were assembled with Short Oligonucleotide Analysis Package (SOAP)denovo (v1.6) into 175 contigs and 33 scaffolds. The *N*_50_ of the assembled scaffolds is 2,712,414 bp and the G+C content is 50.37%. Total assembled bases were 5,221,777 bp, including 122,559 bp of unknown bases (gap). We applied Glimmer v3.0 to predict putative open reading frames on scaffolds (9). The resulting coding sequences (CDSs) were annotated by alignment to the nonredundant (NR), Clusters of Orthologous Groups (COG), and Kyoto Encyclopedia of Genes and Genomes (KEGG) databases. We also aligned CDSs to the Virulence Factors of Pathogenic Bacteria Database (VFDB) and the Antibiotic Resistance Genes Database (ARDB) to detect virulence genes and antibiotic genes (10, 11). Tandem repeat sequences were identified with Tandem Repeats Finder (TRF) v4.04, and scattered repeat sequences were identified with RepeatMasker v3.2.9. We also predicted rRNAs and tRNAs by using RNAmmer and tRNAscan-SE1.21, respectively (12, 13).

### Nucleotide sequence accession number. 

This whole-genome sequence of *E. coli* LCT-EC59 has been deposited at DDBJ/EMBL/GenBank under the accession no. ANHV00000000.
